# Health-Related Quality of Life and Related Factors among Primary Caregivers of Children with Disabilities in Shanghai, China: A Cross-Sectional Study

**DOI:** 10.3390/ijerph17249299

**Published:** 2020-12-12

**Authors:** Cong Xia, Mei Sun, Xinying Li, Chenhao Lu, Xiu Gao, Jun Lu, Gang Chen

**Affiliations:** 1 School of Public Health, Fudan University, Shanghai 200032, China; 19111020035@fudan.edu.cn (C.X.); sunmei@fudan.edu.cn (M.S.); 17211020033@fudan.edu.cn (X.L.); xiugao16@fudan.edu.cn (X.G.); 2 China Research Center on Disability, Fudan University, Shanghai 200032, China; 3 Rollins School of Public Health, Emory University, Atlanta, GA 30322, USA; garylu32@gmail.com

**Keywords:** caregivers, children with disabilities, health-related quality of life, determinants

## Abstract

Health-related quality of life (HRQOL) of caregivers of children with disabilities (CWD) is important for both children’s rehabilitation and caregivers’ life, but the corresponding attention is far from enough in mainland China. Thus, we investigated the HRQOL of 170 caregivers and related factors in Shanghai. The 12-item Short Form Health Survey (SF-12) was used to measure HRQOL. The potential factors were collected, including child characteristics, caregiver characteristics, and environmental factors. Univariate analysis and multiple linear regression were performed to identify the key factors that could be intervened. Compared with the general population, caregivers of CWD had a slightly higher score on the physical component summary (PCS, 52.57 ± 8.41), but the score of mental component summary (MCS, 31.58 ± 7.72) was extremely low. Caregiver’s illness condition, family size, and household income were significant factors of physical HRQOL. Caregivers with illness and caregivers living in an extended family were associated with higher mental HRQOL. Whereas these two factors had opposite effects on physical HRQOL. This finding indicated poor mental HRQOL among caregivers of CWD in Shanghai and thus requiring urgent attention and intervention. Improving physical fitness, maintaining family integration, and providing financial support should be considered when developing intervention for this population.

## 1. Introduction

A caregiver is defined as a person who provides care to those who have difficulties in completing the tasks of daily living and thus need supervision or assistance due to some form of illness or disability [1]. They may provide the care in an institution or an organization, which is called formal caregiving. The concept also refers to an informal caregiver who has a social relationship with the care recipient including family member, relative, friend, or neighbor. With the rapid growth of the global economy, institutionalized long-term care may be an option for children with disabilities (CWD), but the informal caregivers are preferable because of children’s high dependency and cognitive deficiency. Therefore, the caregivers of CWD generally refer to informal caregivers especially family caregivers including parents, grandparents, siblings, or other family members. In recent years, there has been a tremendous change in the health care system which exerts a shift toward family-centered services. This shift highlights the primary and important role of the family in children’s caring and thus increases the responsibilities of informal caregivers [2]. In addition, their contribution on children’s home-based rehabilitation and maintenance of rehabilitation effect can be increasingly enormous [3].

The World Health Organization has reported that around 15% of the world population (one billion people) live with some form of disability and 95 million are children (aged 0–14) [4]. The total number of CWD presents the rising trend, which aggravates the informal caregiver’s childcare burden. Compared with the general population, previous studies have found that caregivers of CWD experienced high stress [5], depression, anxiety, and low satisfaction with life [6,7], resulting in a detrimental effect on their health-related quality of life (HRQOL). In China, the investigated number of CWD in 2006 was 3.87 million, roughly 4.66% of the total population with disabilities [8]. Additionally, caregivers, especially mothers, may feel guilt or remorse for having a child with disability and thus would do their utmost to care for the disabled child alone [9]. These factors will further worsen HRQOL of this special population and therefore should urgently be concerned. However, the current corresponding attention is far from enough.

To our knowledge, there is a wealth of research on the HRQOL of caregivers of CWD abroad, and the relevant literature has mentioned many influencing factors [10,11], including the disability types and behavioral problems of children [12,13,14,15], caregivers’ employment status [16], social participation [17], parental stress [5], and coping strategies [18]. In recent years, domestic preliminary explorations have begun in Taiwan [19,20] and the underdeveloped central and western areas of the mainland, such as Anhui and Hunan [9,21], but neglecting developed areas, especially Shanghai. Comparing the results of existing studies, factors affecting the HRQOL of caregivers of CWD vary with the level of economic development, culture, social support, and other circumstances [22,23,24]. The key and intervening factors also change, suggesting that the results of existing studies do not necessarily apply to caregivers of CWD in Shanghai.

Moreover, with the advances of rehabilitation technology and the support of rehabilitation-related policies, CWD have more rehabilitation and development possibilities, which shift the pressure for rehabilitation from external factors to internal factors for caregivers. This change not only increases the pressure on caregivers to enable their children to receive rehabilitation services, but also reduces opportunities for caregivers’ self-development. Both could lower caregivers’ HRQOL. Shanghai, as an international metropolis, is prosperous in economy and is at the forefront of rehabilitation services for CWD in China. Therefore, it is crucial to explore the HRQOL among caregivers of CWD. Furthermore, the findings could provide references for some developed countries and other similar areas in developing countries.

The International Classification of Functioning, Disability and Health (ICF), which was approved in May 2001 by the World Health Assembly, is a universal framework of functioning and health [25]. It is organized in two parts, one part includes body functions and structures, activities and participation, the other part encompasses contextual factors [26]. ICF emphasizes the dynamic interaction between health condition and personal, environmental factors, which means any factors of caregivers, children, and the environment may have impact on caregiver’s HRQOL. In October 2007, the International Classification of Functioning, Disability and Health—Child and Youth (ICF-CY) was published, and was designed specifically for children and youths [27]. Both ICF and ICF-CY provide a scientific basis for identifying the potential determinants of the caregivers’ HRQOL of CWD comprehensively.

Therefore, the purpose of this study was to measure the HRQOL of caregivers of CWD in Shanghai and to explore the potential influencing factors associated with HRQOL among these caregivers under the guidance of ICF and ICF-CY. Ultimately, the findings could provide references for local health care policymakers and public health researchers to design appropriate health management strategies for the vulnerable population.

## 2. Materials and Methods

### 2.1. Participants and Procedure

We recruited the caregivers of CWD from one district of Shanghai. This district has a well-constructed rehabilitation service system and high-level government attention to CWD. Since the exact number of CWD is not yet available, we determined the sample size based on the number of children who received the Sunshine Baby Cards. The Sunshine Baby Card is the most important project of rehabilitation service system provided for CWD by the Shanghai Health Bureau, Shanghai Municipal Education Commission, Shanghai Finance Bureau, and the Shanghai Disabled Person’s Federation. It can be applied for any child who has been certified as disabled by a professional organization. According to the regulation, every child who holds a Sunshine Baby Card can receive rehabilitation training in designated rehabilitation institutions and enjoy the corresponding subsidies. Thus, the number of children with a Sunshine Baby Card approximates the actual number of CWD. In 2019, there were 900 children with the Sunshine Baby Card in the district, 15% of the total number of cardholders were selected for the survey. Then the sample size was expanded by 20% to take into account the dropout rate, which resulted in a sample size of 900 × 15% × (1 + 20%) = 162. Finally, 170 cases were included in the survey.

We collected data in both rehabilitation institutions and community-based settings, considering that children aged 0–6 years are more accessible in rehabilitation institutions, while children aged ≥7 years need to go to school. Since there are private rehabilitation institutions, public hospitals, and community health centers in Shanghai, we selected all types of rehabilitation institutions for investigation. For community-based settings, we covered all communities of this district.

Caregivers were included if (1) they were caring for a child diagnosed with disabilities by the responsible departments; (2) their children were aged 0–16 years; and (3) they were the primary caregivers. Caregivers who did not identify themselves as primary caregivers were excluded from the study. In each rehabilitation institution, the children’s basic information and rehabilitation schedule was first obtained from their health records to determine whether they met the criteria or not. Then, we created the investigation list. As for the communities, we first collected the basic information of CWD from the staff of the communities. If children met the sample requirements, their name would be fed back to the staff. The purpose and procedure of the research project were explained to caregivers. If they agreed to participate in the study, the investigators would conduct a one-to-one investigation. Ultimately, we recruited 97 caregivers from the rehabilitation institutions and 73 caregivers from the communities.

Ethics approval was obtained from the Ethics Committees of School of Public Health of Fudan University (Grant No. IRB#2019-10-0782).

### 2.2. Measures

#### 2.2.1. Measure of Health-Related Quality of Life

HRQOL was measured using the 12-item Short Form Health Survey (SF-12), an abbreviated form of MOS 36-item Short Form Health Survey (SF-36). SF-12 was designed to take less time to complete and was widely used due to its high degree of acceptability and data quality. It contains 8 domains: Physical Functioning (PF), Role-Physical (RP), Bodily Pain (BP), General Health (GH), Energy/Fatigue (VT), Social Functioning (SF), Role-Emotional (RE), and Mental Health (MH) [28]. Two summary scores are reported: Physical Component Summary (PCS) score and Mental Component Summary (MCS) score which can be used to measure physical HRQOL and mental HRQOL, respectively. For comparison, scores were calculated using the US norm-based scoring algorithm. The test–retest reliability (0.89) and reliability coefficients (0.76) showed the scale was reliable and valid [28]. In 2005, a study conducted in Hong Kong using SF-12 showed that this instrument was valid and equivalent for the Chinese population as well as the items and scoring algorithm. Therefore, SF-12 can be applied to the Chinese population for further health-related research [29].

#### 2.2.2. Measure of Determinants of HRQOL

Child characteristics: Child characteristics were designed under the framework of ICF-CY. For body functions and structures, sleep time and emotional stability were taken into consideration. For activities and participation, we measured the frequency of physical activities and group games during the last four weeks. For personal factors, we collected age, gender, cause of disability, disability type, disability severity, and whether they had the Sunshine Baby Card or disability certificate.

According to the People with Disabilities Act of the Peoples Republic of China, disability is divided into visual disability, hearing disability, speech disability, physical disability, intellectual disability, mental disability, multiple disability, and other disabilities. Since our inclusion criteria did not strictly limit the type of disability, all types above were involved in our objects. Additionally, we specially listed cerebral palsy as one specific type of disability because of its high prevalence among children. Disability severity was graded with China’s legal criteria of disability which divided all kinds of disabilities into four levels, ranging from level I (extremely severe) to IV (mild severe).

Caregiver characteristics: Caregiver characteristics were collected under the guidance of ICF. The dimension of body function and structure was measured with the question of “whether you have the following disease or not (e.g., hypertension, diabetes, Parkinson’s disease)”. The feature of activity and participation was identified by caregivers’ employment status and caring time. Furthermore, we collected the personal factors including relationship with the child, gender, household register, marital status, and education. Previous studies mostly focused on the caregiving of parents or merely mothers, rarely involving other families. In this study, we took fathers, mothers, paternal grandfathers, paternal grandmothers, maternal grandfathers, and maternal grandmothers into consideration.

Environmental factors: Environmental factors included number of children, family size, household income, policy satisfaction, and social friendliness. Among these factors, policy satisfaction and social friendliness were reported by subjective perception of caregivers and were evaluated in the form of the Likert scale, ranging from 1 (not friendly at all or dissatisfaction) to 5 (extremely friendly or satisfaction).

### 2.3. Statistical Analysis

Descriptive statistical methods were applied in presenting basic characteristics for child, caregiver, and environment, and the measures (mean, standard deviation [SD], frequencies, and percentages) were respectively reported. Mean scores of the SF-12 of caregivers of CWD were compared with norm scores of the general population using the one-sample *t*-test. In order to preliminarily examine the associations between participants’ characteristics and HRQOL, univariate analyses including *t*-test and one-way analysis of variance (ANOVA) were conducted. To further investigate the potential predictors, two multiple linear regressions were performed with PCS and MCS scores as dependent variables. Any covariates that achieved *p* < 0.10 for the bivariate analyses were included in the multivariate linear regression models. We examined the multicollinearity among predictors in each model using Variance Inflation Factor (VIF). We removed the predictors which lead to multicollinearity. All statistical analyses were performed using SPSS Statistics version 20.0 (IBM Corp., Armonk, New York, NY, USA).

## 3. Results

### 3.1. Descriptive Statistics

A total of 170 caregivers completed the survey. The mean age of children was 7.16 years (SD: 4.04 years), 60.6% of the children were male, and most of them (62.4%) got disabled because of acquired disease, trauma, or unknown reason. Both children with cerebral palsy and children with physical disability had the highest percent (20.0%). Moreover, the disability severity was mostly unrated (70.6%) (Table 1). Table 2 and Table 3 illustrate the caregiver characteristics and environmental factors. The mean age of the caregivers was 45.10 years (SD: 12.16 years). The relationship with the child was mother (48.2%), father (25.3%), and grandparents (25.9%). A total of 65.9% of the caregivers were female and most of them were currently married (91.2%). In addition, 78.8% of the disabled children were only children and most of the families were small families with no more than four persons.

### 3.2. HRQOL

Table 4 shows the scores on HRQOL. The mean PCS score was 52.57 (SD: 8.41) and the mean MCS score was 31.58 (SD: 7.72). Compared to mothers, fathers had the highest scores on PCS (55.59 ± 5.89), but the lowest scores on MCS (30.67 ± 6.29). For disability types, caregivers of children with hearing disabilities had the lowest scores on PCS (45.77 ± 13.23), and caregivers of children with cerebral palsy had the lowest scores on MCS (29.25 ± 6.43). Moreover, compared with the Chinese (HK) and the US norm of the general population, caregivers of CWD had significantly higher mean scores on PCS and lower mean scores on MCS (*p* < 0.001) (Table 5) [28,29].

### 3.3. Univariate Analysis

Results of the univariate analysis using the PCS and MCS of the caregivers as the dependent variables and 24 factors are shown in Table 6. In summary, among 11 factors associated with CWD, the physical HRQOL of caregivers were significantly different in age, disability certificate, disability severity, sleep time, and emotional stability (*p* < 0.10). However, the mental HRQOL of caregivers was found as the only statistically significant difference in disability type (*p* < 0.10). For the eight factors associated with caregivers, statistically significant differences in the physical HRQOL were found in role, gender, education, employment status, and accompanying disease (*p* < 0.10). Statistically significant difference in the mental HRQOL was found in caregivers’ accompanying disease (*p* < 0.10). For the five environmental factors, the physical HRQOL of caregivers were significantly different in family size, household income, and policy satisfaction. Whereas statistically significant differences in mental HRQOL were found in number of children, family size, and policy satisfaction (*p* < 0.10).

### 3.4. Multiple Linear Regression

The two fitted multiple regression models identified predictors of caregivers HRQOL as measured with the SF-12 (Table 7 and Table 8). PCS (or MCS) score was used as the dependent variable, and the significant factors in univariate analysis were included as independent variables. We examined the severity of multicollinearity in the multiple linear regression models using Variance Inflation Factor (VIF). Therefore, we removed the disability certificate because of the high VIF (7.937 > 5). The PCS regression model showed that caregiver’s illness condition, family size, and household income had a statistically significant effect on the physical HRQOL of caregivers of CWD. Specifically, caregivers with illness (*β* = −0.291, *p* < 0.001) and caregivers living in an extended family with more than six persons (*β* = −0.165, *p* = 0.032) had lower physical HRQOL. Household income was positively correlated with the physical HRQOL of caregivers. The MCS regression model showed that caregiver’s illness condition and family size were statistically significant factors, indicating that caregivers had higher mental HRQOL when they had an accompanying illness (*β* = 0.286, *p* = 0.005) or the household’s resident population was six or more (*β* = 0.216, *p* = 0.003).

## 4. Discussion

### 4.1. Exploring Factors Affecting the HRQOL of Caregivers in Shanghai has Important Value for the Rehabilitation of CWD

Our research group was dedicated to the disability prevention, rehabilitation, and health of CWD for a long time. HRQOL, as the health aspect of quality of life, focuses on people’s level of ability, daily functioning, and ability to experience a fulfilling life. Whereas caregivers’ HRQOL may not only affect their own living standard, but also has significant impact on the work productivity which is the guarantee for adequate care and caregiving quality. Moreover, previous studies also demonstrated significant correlations between caregivers’ HRQOL and the rehabilitation effect as well as community reintegration of CWD [3,30]. Therefore, exploring the facilitators and barriers affecting the caregivers’ HRQOL and identifying the key factors can provide guidance for implementing targeted interventions. They are also valuable for promoting the rehabilitation of CWD and improving their health.

### 4.2. Parents Are the Primary Caregivers of CWD, but the Caregiving Role of Grandparents Cannot be Ignored

Among the 170 caregivers of CWD, most of them were parents (73.5%) with the highest proportion of mothers (48.2%), which is consistent with most studies [31,32]. It is worth noting that the proportion of grandparents in this study is 25.9%, reflecting the social phenomenon of the elderly bringing up their grandchildren. In fact, raising grandchildren by the elderly is not a unique scenery in China, as the elderly in many countries are on the road to bring up grandchildren, and even become the main force. In Europe, 58% of grandmothers and 49% of grandfathers took care of at least one of their grandchildren in the preceding year [33]. In the United States, 5.7 million grandparents lived with their grandchildren in 2000, and the numbers seemed to be on the rise [33]. In 2002, 60–70% of Taiwanese elderly lived with their married children or with grandchildren [34]. For families with CWD, it is important to have two earners that maintain the functioning of the families. Neither mothers nor fathers may be in a position to provide the desired amount of childcare inside the nuclear household. According to a carer survey in urban Britain, 79% of the respondents were predominantly grandparents [35]. In this study, we found that grandparents (51.81 ± 9.92) had lower scores on physical HRQOL than parents (52.77 ± 7.83) because of the physical degeneration of elders. Thus, intervention strategies should cover not only parents but also grandparents.

### 4.3. Physical HRQOL of Caregivers of CWD is Moderate, But the Mental HRQOL Needs to Be Urgently Concerned

The results of the normative comparison showed that the physical HRQOL of caregivers was better than that of the general population [28,29]. Possible explanations for this phenomenon are that 73.53% of the study population were parents, while the normative model consisted of the entire population. In addition, there is an adverse selection tendency to choose the person with better HRQOL as the primary caregiver in order to ensure the caregiving quality for the disabled children.

Meanwhile, we found that the mental HRQOL of caregivers of CWD in this study (31.58 ± 7.72) was worse than that of the general population. This finding is consistent with other studies [36], but the mental condition among caregivers in our study was extremely poor. Actually, this result validates our previous hypothesis. In developing countries, there are substantial shortfalls in the availability of rehabilitation services, and the effectiveness of rehabilitation might not be satisfactory [37,38]. Insufficient social inclusion in these places also makes it difficult for CWD to attend school [39]. The United Nations Children’s Fund estimates that 90% of CWD in Africa do not attend school and are thus less likely to engage in other opportunities for social participation [40,41]. Furthermore, living in developed cities like Shanghai, the high living expense often puts great financial pressure on caregivers, especially those who have CWD. In addition, poor mental HRQOL may be also associated with China’s one-child policy, which had been implemented for 30 years and caused the prevalence of the only-child family [42]. On one hand, if parents were the only child, their capability of adapting themselves to the adversity was relatively weak. On the other hand, if the child with a disability is also an only child, the psychological trauma to the parents is conceivable. All of these issues could increase parental concern and aggravate caregivers’ psychological status. Therefore, relevant government departments and community committees should pay more attention to this group and provide corresponding social support.

### 4.4. The Disease Condition of Caregiver Has a Negative Effect on Physical HRQOL But a Protective Effect on Mental HRQOL

Disease condition was found to have a significant impact on both the physical and mental HRQOL of caregivers. Specifically, caregivers with a disease had a worse physical HRQOL than those without illness, which is consistent with some previous research [36]. However, several studies found no statistical difference in physical and mental HRQOL between caregivers with and without disease [43]. In this study, 34.7% of the caregivers were found to have one or more diseases, including hypertension (15.3%), arthritis/rheumatic disease (5.3%), diabetes (4.7%), and coronary heart disease (3.5%). This is consistent with a Canadian study [30] that found that caregivers of children with cerebral palsy had significantly higher rates of hypertension, diabetes, and migraines than ordinary caregivers. Both findings suggest that caregivers should be concerned about their disease condition and be provided with timely and targeted professional support, for example, physical examination and medical assistance service. Additionally, the psychological HRQOL of the caregivers with disease was better than those without disease. This difference is beyond our expectation, but in fact is a common psychological phenomenon. Caregivers with a disease have lower caregiving expectations which increases the feasibility of achieving the goal. Therefore, there is rising satisfaction and higher mental HRQOL.

### 4.5. Family Size Expansion Benefits the Mental HRQOL of Caregivers, But Often Means a Greater Need for Physical Assistance and Support

Different from our common sense, this study found that caregivers had better mental HRQOL but worse physical HRQOL when they lived in a family with the permanent residents of six or more, compared with those who lived with less than two persons. With the development of urbanization, the family size tends to be smaller and simpler, with the nuclear family becoming the mainstream family form in modern society [44]. It is a well-documented phenomenon that women’s fertility intention is low or falling rapidly everywhere except in parts of Sub-Saharan Africa [45,46]. The more developed the region, the more pronounced this phenomenon is [47]. In these places, older people emphasize fostering independence in their children, family integration is unlikely to occur if the nuclear family could withstand the caregiving pressure. However, if the parents are unable to cope with the corresponding stress because of poor physical HRQOL, they would likely turn to the child’s grandparents in order to increase the overall resilience [48]. Meanwhile, there was high consistency in independent views of mothers and fathers of grandparents identified by both as the most available and supportive [49]. Therefore, the family size expands.

In addition, it has been confirmed by several studies [50,51,52] that social support (including family support, and support from friends) can positively influence the mental HRQOL of caregivers. The corresponding plausible explanation is that other family members are able to provide emotional support to caregivers when there are more persons in the family. Furthermore, the psychological stress of caregivers is reduced. Therefore, relevant departments should strengthen humanistic care for caregivers with small families, providing psychological support such as door-to-door communication and psychological counseling.

### 4.6. Caregivers of Children with Disabilities Face Tremendous Financial Pressures, Providing Financial Support Could Improve Their HRQOL

In this study, we found that household income was significantly positive associated with the physical HRQOL of caregivers. Higher income could improve caregivers’ parental physical environment and increase the access to health services [53]. This finding is consistent with most previous studies. In fact, for all families of CWD, caregivers are under tremendous financial pressure [23]. On the one hand, providing the basic necessities and rehabilitation for these children is costly. On the other hand, caregivers often have to quit their jobs to take care of the child full time, which reduces their source of income [54]. However, we did not find a statistically significant effect of household income on the mental HRQOL of caregivers. This is mainly because the household income of families with CWD is always insufficient compared with the child’s rehabilitation expense, and financial need is always the most pressing. This finding suggests that although some financial subsidies are already available, they are far from enough and need to be further adjusted. Therefore, relevant government departments could provide additional financial support depending on families’ economic status, and this might be useful in enhancing the HRQOL of caregivers and their children.

### 4.7. Implications and Limitations

Results from this study have practical implications for health professionals and policymakers to help improve the HRQOL of caregivers of CWD. We provide three suggestions as follows. First, rehabilitation physicians should pay attention to the mental health of caregivers and regularly conduct psychological counseling to reduce their psychological pressure; second, local governments should understand the comprehensive and dynamic information about the caregivers, their children, and living environment, so as to provide targeted physical examination, medical assistance service, humanistic care, and financial support; finally, government departments should improve the effectiveness of services for CWD and provide coverage for them. After all, as long as the child is healthy, the caregiver is better off.

There are several limitations in this study. Firstly, the study design was cross-sectional. Thus, it was difficult to establish causal relationships between caregivers’ HRQOL and related factors. Secondly, caregivers of children with all types of disabilities were included in this study, therefore it was hard to identity the determinants of caregivers’ HRQOL of children with specific disability. Thirdly, the sample size is only 170, which may affect the accuracy of the HRQOL levels. Finally, we include many factors that could influence caregivers HRQOL, but these factors were measured crudely, such as using just one question. Actually, this is a preliminary exploration to discover significant factors, and more in-depth studies will be followed up based on this research.

## 5. Conclusions

In summary, this study found poor mental HRQOL among caregivers of CWD in Shanghai, and thus requiring urgent attention and intervention. The factors we identified provide an intervention framework for promoting caregivers’ HRQOL from the perspective of children, caregivers, and the environment. Significant factors indicate the key role of caregiver’s illness condition, family size, and household income. Therefore, interventions should be targeted at these key factors to improve HRQOL of caregivers and further facilitate children’s rehabilitation and health. Additionally, it is worth noting for local health care policymakers and public health researchers that relevant health management strategies should not only consider parents but also grandparents.

## Figures and Tables

**Table 1:** Descriptive statistics of the characteristics of the children with disabilities (*N* = 170).

Characteristics	*N* (%)	Mean (SD)
Age (years)		7.16 (4.04)
Age group (years)		
[0, 3)	15 (8.8)	
[3, 6)	58 (34.1)	
[6, 12)	65 (38.2)	
[12, 16]	32 (18.8)	
Gender		
Male	103 (60.6)	
Female	67 (39.4)	
Cause of disability		
Congenital	64 (37.6)	
Acquired	106 (62.4)	
Sunshine Baby Card		
Yes	146 (85.9)	
No	24 (14.1)	
Disability Certificate		
Yes	48 (28.2)	
No	122 (71.8)	
Disability type		
Vision	18 (10.6)	
Hearing	4 (2.4)	
Speech	18 (10.6)	
Physical	34 (20.0)	
Cerebral palsy	34 (20.0)	
Intellectual	21 (12.4)	
Mental	14 (8.2)	
Multiple disabilities	27 (15.9)	
Chinese standard for categorizing disability severity		
I&II	41 (24.1)	
III&IV	9 (5.3)	
Unrated	120 (70.6)	
Sleep time per day (hours)		
0–7	19 (11.2)	
8–10	117 (68.8)	
≥11	34 (20.0)	
Emotional stability		
Unstable	37 (21.8)	
Fair	35 (20.6)	
Very stable	63 (37.1)	
Extremely stable	35 (20.6)	
Physical activities (times/month)		
0	86 (50.6)	
1–2	29 (17.1)	
3–4	17 (10.0)	
≥5	38 (22.4)	
Group games (times/month)		
0	62 (36.5)	
1–2	39 (22.9)	
3–4	20 (11.8)	
≥5	49 (28.8)	

**Table 2:** Descriptive statistics of caregivers’ characteristics of children with disabilities (*N* = 170).

Characteristics	*N* (%)	Mean (SD)
Age (years)		45.10 (12.16)
Relationship with the child ^a^		
Father	43 (25.3)	
Mather	82 (48.2)	
Grandparents	44 (25.9)	
Gender		
Male	58 (34.1)	
Female	112 (65.9)	
Household register		
Shanghai	138 (81.2)	
Others	32 (18.8)	
Marital status		
Married	155 (91.2)	
Divorced or widowed	15 (8.8)	
Education		
Junior high school and below	26 (15.3)	
Senior high school or technical secondary school	45 (26.5)	
Junior college	33 (19.4)	
Bachelor or above	66 (38.8)	
Employment status		
Full-time	84 (49.4)	
Part-time	23 (13.5)	
No job	63 (37.1)	
Disease		
Yes	59 (34.7)	
No	111 (65.3)	
Caregiving time (years) ^b^		
[0, 5]	78 (45.9)	
(5, 10)	59 (34.7)	
(10, 18)	29 (17.1)	

^a^ one datum is the nanny; ^b^ four dates missing.

**Table 3:** Descriptive statistics of the environmental factors (*N* = 170).

Characteristics	*N* (%)	Mean (SD)
Number of children		1.22 (0.45)
1	134 (78.8)	
≥2	36 (21.2)	
Number of permanent residents in a household		3.98 (1.13)
≤3	74 (43.5)	
4	48 (28.2)	
5	30 (17.6)	
≥6	18 (10.6)	
Household income monthly (¥) ^a^		
0–10,000	54 (31.8)	
10,001–15,000	34 (20.0)	
15,001–20,000	38 (22.4)	
>20,000	40 (23.5)	
Policy satisfaction		
Dissatisfaction	45 (26.5)	
Moderately satisfaction	85 (50.0)	
Very or extremely satisfaction	40 (23.5)	
Social friendliness		
Extremely	12 (7.1)	
Quite a bit	48 (28.2)	
Moderately	70 (41.2)	
A little bit or not at all	40 (23.5)	

^a^ four dates missing.

**Table 4:** The 12-item Short Form Health Survey (SF-12) mean scores of caregivers under different disability types of children and relationship with them (*N* = 170).

SF-12	Disability Type	Caregivers Relationship with the Child ^a^			Total
		Mother (*N* = 82)	Father (*N* = 43)	Grandparents (*N* = 44)	
		Mean (SD)/Median	Mean (SD)/Median	Mean (SD)/Median	
PCS		51.29 (8.34)	55.59 (5.89)	51.81 (9.92)	52.57 (8.41)
	Vision	53.04 (5.31)	54.17 (6.33)	55.79 (4.33)	54.15 (5.40)
	Hearing	42.73 (16.77)	38.64 (0.00)	58.97 (0.00)	45.77 (13.23)
	Speech	54.14 (6.64)	60.21 (2.41)	56.47 (9.49)	55.46 (6.73)
	Physical	51.75 (8.61)	58.12 (3.47)	50.24 (6.08)	53.36 (7.45)
	Cerebral palsy	51.73 (6.72)	55.49 (4.37)	52.71 (11.39)	53.21 (8.28)
	Intellectual	48.80 (10.61)	52.59 (0.55)	50.55 (5.44)	49.75 (8.51)
	Mental	49.78 (9.64)	56.99 (6.42)	52.84 (10.79)	52.72 (9.04)
	Multiple disabilities	51.04 (8.35)	53.71 (8.42)	45.18 (17.66)	50.82 (10.53)
MCS		31.55 (7.14)	30.67 (6.29)	32.72 (9.77)	31.58 (7.72)
	Vision	32.55 (5.29)	30.12 (5.82)	26.08 (1.26)	30.03 (5.32)
	Hearing	37.36 (3.27)	32.33 (0.00)	25.48 (0.00)	33.13 (5.93)
	Speech	34.23 (10.23)	27.05 (3.87)	27.93 (3.64)	32.10 (9.13)
	Physical	31.20 (8.02)	32.14 (5.08)	41.50 (10.04)	33.67 (8.58)
	Cerebral palsy	29.32 (3.44)	28.59 (7.46)	29.69 (7.84)	29.25 (6.43)
	Intellectual	33.35 (8.00)	39.94 (9.75)	39.66 (11.48)	36.08 (9.48)
	Mental	29.74 (7.19)	30.03 (1.79)	27.90 (7.49)	29.30 (5.87)
	Multiple disabilities	29.49 (4.69)	31.45 (8.22)	32.10 (9.78)	30.10 (6.40)

PCS, physical component summary score; MCS, mental component summary score. ^a^ one datum is the nanny, the PCS was 60.98, and the MCS was 23.17.

**Table 5:** Comparisons of SF-12 mean scores between caregivers of children with disabilities (CWD) and Chinese (HK) norm and US norm.

SF-12	Caregiver Mean (SD)	Chinese (HK) Mean (SD)	US Mean (SD)
PCS	52.57 (8.41)	50.0 (9.4) **** 8	50.12 (9.45) ****
MCS	31.58 (7.72)	50.0 (9.5) ****	50.04 (9.59) ****

*p* values are from one-sample *t*-test. **** *p* < 0.001.

**Table 6:** Univariate analysis of influencing factors on the health-related quality of life (HRQOL) of caregivers of CWD (*N* = 170).

Factors	PCS		MCS	
	Statistic	*p* Value	Statistic	*p* Value
Children characteristics				
Age group	3.572	0.015 **	1.167 ^b^	0.328
Gender	0.651	0.516	0.507	0.613
Cause of disability	1.139	0.256	−0.942	0.347
Sunshine Baby Card	−0.620	0.536	−0.370	0.712
Disability Certificate	−2.441 ^a^	0.017 **	0.848	0.398
Disability type	1.386	0.215	2.395	0.023 **
Disability severity	4.482 ^b^	0.016 **	0.331	0.719
Sleep time	5.403 ^b^	0.008 ***	1.097 ^b^	0.341
Emotional stability	2.826	0.041 **	0.431	0.731
Physical activities	1.555	0.202	0.359	0.783
Group games	1.484	0.221	1.182 ^b^	0.320
Caregivers characteristics				
Relationship with the child	4.112 ^b^	0.019 **	0.732 ^b^	0.483
Gender	3.072 ^a^	0.003 ***	−1.146	0.253
Household register	−0.758	0.450	0.616	0.538
Marital status	1.134 ^a^	0.274	−0.093	0.926
Education	2.352	0.074 *	0.733 ^b^	0.535
Employment status	5.774 ^b^	0.019 **	2.128	0.122
Disease	−4.471 ^a^	<0.001 ****	3.609	<0.001 ****
Caregiving time	1.887	0.155	0.388 ^b^	0.680
Environmental factors				
Number of children	1.209	0.228	−1.786	0.076 *
Family size	3.049	0.030 **	6.980	<0.001 ****
Total household income	3.503 ^b^	0.017 **	0.819	0.485
Policy satisfaction	3.171 ^b^	0.046 **	3.767	0.025 **
Social friendliness	1.552	0.203	0.251 ^b^	0.860

^a^ Equal variances not assumed, *t*’ test was used; ^b^ equal variances not assumed, Brown–Forsythe test was used. * *p* < 0.1; ** *p* < 0.05; *** *p* < 0.01; **** *p* < 0.001.

**Table 7:** Multiple regression models for physical HRQOL of caregivers of CWD (*N* = 170).

	Control	B	SE	*β*	*p* Value
Child’s age	[0, 3)				
[3, 6)		0.369	2.311	0.021	0.873
[6, 12)		0.044	2.502	0.003	0.986
[12, 16]		−1.714	3.001	−0.080	0.569
Disability degree	Unrated				
I&II		−0.763	1.632	−0.039	0.641
III&IV		−1.696	2.783	−0.045	0.543
Child’s sleep time per day	≤7 h				
8–10 h		3.457	2.201	0.191	0.118
≥11 h		3.675	2.674	0.175	0.171
Child’s emotional stability	Unstable				
Fair		0.913	1.889	0.044	0.630
Very stable		1.450	1.636	0.084	0.377
Extremely stable		2.340	1.920	0.113	0.225
Caregiver’s relationship with the child	Grandparents				
Father		0.039	2.622	0.002	0.988
Mather		0.625	1.990	0.037	0.754
Caregiver’s gender	Male				
Female		−3.701	2.497	−0.209	0.140
Caregiver’s education	Junior high school and below				
Senior high school/technical secondary school		2.912	2.021	0.153	0.152
Junior college		−1.531	2.238	−0.072	0.495
Bachelor or above		−0.105	2.222	−0.006	0.962
Caregiver’s employment status	No job				
Full-time		−0.922	1.679	−0.055	0.584
Part-time		−2.402	2.034	−0.098	0.240
Disease condition	No disease				
One or more diseases		−5.117	1.266	−0.291	<0.001 ****
Family size	≤3				
4		0.079	1.494	0.004	0.958
5		2.160	1.657	0.098	0.195
≥6		−4.486	2.070	−0.165	0.032 **
Total household income monthly	0–10,000 (¥)				
10,001–15,000		2.267	1.618	0.108	0.163
15,001–20,000		3.691	1.626	0.183	0.025 **
>20,000		4.262	1.619	0.216	0.009 ***
Policy satisfaction	Dissatisfaction				
Moderately satisfaction		2.014	1.494	0.120	0.180
Very or extremely satisfaction		2.865	1.734	0.145	0.101

R² = 0.630, F = 3.457, *p* < 0.001. ** *p* < 0.05; *** *p* < 0.01; **** *p* < 0.001.

**Table 8:** Multiple regression models for physical mental HRQOL of caregivers of CWD (*N* = 170).

	Control	B	SE	*β*	*p* Value
Disability type	Multiple disabilities				
Vision		1.438	3.928	0.028	0.715
Hearing		2.983	2.306	0.122	0.198
Speech		3.280	2.077	0.169	0.116
Physical		−1.473	2.086	−0.077	0.481
Cerebral palsy		3.334	2.304	0.143	0.150
Intellectual		−1.398	2.496	−0.050	0.576
Mental		−0.996	2.157	−0.047	0.645
Number of children	1				
≥2		−0.074	1.762	−0.004	0.967
Disease condition	No disease				
One or more diseases		3.490	1.173	0.216	0.003 ***
Family size	≤3				
4		0.957	1.401	0.056	0.496
5		−1.316	1.634	−0.065	0.422
≥6		7.147	2.494	0.286	0.005 ***
Policy satisfaction	Dissatisfaction				
Moderately satisfaction		−2.476	1.324	−0.161	0.063
Very or extremely satisfaction		−0.521	1.591	−0.029	0.744

R² = 0.515, F = 3.998, *p* < 0.001. *** *p* < 0.01.
